# METACHRONOUS COLORECTAL LIVER METASTASIS

**DOI:** 10.1590/0102-6720202500005e1874

**Published:** 2025-04-07

**Authors:** Cássio Virgílio Cavalcante de OLIVEIRA, Rodolfo Carvalho SANTANA, Felipe José Fernandez COIMBRA, Alfred KOW, Timothy M. PAWLIK, Rene ADAM, Olivier SOUBRANE, Paulo HERMAN, Ricardo Lemos COTTA-PEREIRA, International Hepato-Pancreato-Biliary Association

**Affiliations:** 1 Universidade Federal da Paraíba, Surgery Department of Surgery - João Pessoa (PB), Brazil; 2 São Rafael Hospital/Rede D’Or Hospital Group, Department of Surgery of the Upper Digestive System - Salvador (BA), Brazil; 3 A. C. Camargo Cancer Center, Department of Surgical Oncology - São Paulo (SP), Brazil; 4 National University Hospital, Department of Surgery, Division of Hepatobiliary and Pancreatic Surgery and Liver Transplantation - Singapore; 5 The Ohio State University, Wexner Medical Center, Department of Surgery - Columbus (OH), USA; 6 University Paris-Saclay, AP-HP Paul Brousse Hospital, Cancer and Transplantation Unit, Hepato Biliary Surgery - Villejuif, France; 7 Universite Paris Descartes, Institute Mutualiste Montsouris, Oncologic and Metabolic Surgery, Department of Digestive - Paris, France; 8 Universidade de São Paulo, Faculty of Medicine, Department of Gastroenterology - São Paulo (SP), Brazil; 9 D’Or Institute for Research and Education, Digestive Surgery Residency Program - Rio de Janeiro (RJ), Brazil

**Keywords:** Colorectal Neoplasms, Neoplasm Metastasis, Liver, Hepatectomy, Neoplasias Colorretais, Metástase Neoplástica, Fígado, Hepatectomia

## Abstract

Deaths related to colorectal cancer are generally associated with its metastases that affect the liver (50%) through the hematogenous route. Approximately 20-25% of these patients already have synchronous metastases in the liver at the time of primary tumor diagnosis. In others, liver metastases will occur during the course of the disease and are called metachronous. Metachronous metastases are believed to have a better prognosis; however, 20-25% of metastatic cases can be resected during the course of the disease. There is a lack of consensus on the diagnostic time interval for metastases to be considered metachronous in the consulted literature. Surgical treatment of metastases and lymph nodes is indicated, and extrahepatic neoplastic disease must be carefully evaluated. Liver transplantation can benefit the patient, should be evaluated, and is indicated in some special situations.

## RESUMO

Os óbitos relacionados ao câncer colorretal são geralmente associadas às suas metástases que afetam o fígado (50%), por via hematogênica. Aproximadamente 20-25% desses pacientes já apresentam metástases sincrônicas no fígado no momento do diagnóstico do tumor primário. Em outros, as metástases hepáticas ocorrerão durante o curso da doença e são chamadas de metacrônicas. Acredita-se que as metástases metacrônicas tenham um melhor prognóstico, no entanto, 20%-25% dos casos metastáticos podem ser ressecados durante o curso da doença. Não há consenso sobre o intervalo de tempo diagnóstico para que uma metástase seja considerada metacrônica na literatura consultada. O tratamento cirúrgico das metástases e dos linfonodos é indicado, e a doença neoplásica extra-hepática deve ser cuidadosamente avaliada. O transplante de fígado pode beneficiar o paciente, deve ser avaliado e é indicado em algumas situações especiais.

## INTRODUCTION

Deaths related to colorectal cancer are generally associated with its metastases that affect the liver (50%) through the hematogenous route^13^ ^,^ ^17^. Approximately 20-25% of these patients already have synchronous metastases in the liver at the time of primary tumor diagnosis. In others, liver metastases will occur during the course of the disease and are called metachronous^13^. Metachronous metastases are believed to have a better prognosis. Only 20-25% of metastatic cases can be resected during the course of the disease^16^.

It is worth mentioning the lack of consensus on the diagnostic time interval for metastases to be considered metachronous since we found articles defining metachronous whose interval between primary diagnosis and the detection of metastatic disease used in the literature ranges from the time to primary resection to 3-6 months.

### When to indicate upfront surgery

In the face of these patients, three situations can be observed:

- Patients with lesions that are certainly unresectable;
- Clearly resectable patients;
- Patients who are probably unresectable, but with potential conversion, where we can include not only the presence of hepatic impairment but also extrahepatic impairment^3^ ^,^ ^15^ ^,^ ^21^.

In the first situation, surgery is already out of the question. In the second, despite the absence of already established criteria, neoadjuvant can be instituted, evaluating the response to chemotherapy, aiming at future strategies, favoring R0 resection, or even the elimination of unobserved micrometastases. Therefore, systemic and hepatic chemotoxicity must always be taken into account. It is worth mentioning that in a meta-analysis of 18 published studies, no benefit was found for these patients^11^. Therefore, in the absence of evidence favoring neoadjuvant therapy or upfront surgery, some authors, such as Adam et al.^2^, defend the concept that technically resectable patients, but with unfavorable oncological criteria, undergo prior chemotherapy.

The search for better results and prognostic criteria that may collaborate with patient selection has become paramount. Fong et al.^7^ published the Clinical Risk Score (CRS), considering the presence of a single nodule, carcinoembryonic antigen (CEA) values, the interval between the diagnosis of the primary lesion and the onset of metastases, its size, and the presence of lymph node involvement at the time of primary resection. The presence of three or more of these criteria indicates a poor prognosis. CRS is criticized for not using histopathological parameters to better predict results^4^, not studying the microenvironment of the tumor/parenchyma interface^8^, or including molecular markers such as KRAS (Kirsten rat sarcoma viral oncogene homolog), NRAS (neuroblastoma RAS viral oncogene), or BRAF (V-Raf Murine Sarcoma Viral Oncogene Homolog B)^15^. These are widely known factors, and according to the affected codon of mutant variables, the prognosis will be worse, not only in overall survival (OS) but also in disease-free survival (DFS) after resection^15^. The mutation finding often suggests a probable focus of metastases, intrahepatic or extrahepatic.

According to Margonis et al.^15^, tumor biology is a key factor in prognostic determination. The R0 margin is not the only beneficial factor for mutant KRAS carriers, as recurrences occur mainly far from metastases, unlike wild-type KRAS, where they occur close to resection. The choice of drugs used in systemic treatment also does not differ from the molecular aspects, and the state of the RAS determines resistance to a series of drugs, guiding the selection of tables, whether adjuvant or neoadjuvant, increasing not only DSF but also 5-year OS^8^.

The lack of robust evidence of prognostic criteria to better select the time, sequence, and size of the resection has generated new studies with the same purpose, taking into account the association between clinical and molecular aspects^9^. In a promising study by Ruzzenente et al.^19^ of patients undergoing prior chemotherapy, they correlated the tumor burden score (TBS)^19^, which uses the combination of the size and the total number of preoperative metastases, with the genetic characteristics of the tumor, KRAS, NRAS, and BRAF wild-type type, against the KRAS, NRAS, and BRAF mutant. They concluded that genetic status was the main prognostic factor in patients with TBS <6. For patients with a genetic status ≥6, only Delta TBS is important, with a response ≥10% after neoadjuvant therapy, which is a sign of a better prognosis^19^ ^,^ ^20^.

In patients that were probably unresectable but with the potential to rescue resectability, a meta-analysis with a systematic review showed a high equivalence between the primary tumor and the metastases^14^, therefore, chemotherapy should be established, respecting selection criteria based on the profile of the primary disease^16^.

### When do extrahepatic metastases contraindicate resection?

In addition to the liver, three other metastatic implant foci can be seen with relative frequency: lung, peritoneum, and lymph nodes^3^.

The lung can be the focus of colorectal metastases in approximately 15% of cases, which may occur in isolation or in association with other foci. When present synchronously with the liver, both resectable, patient survival will be similar to the presence of isolated liver injury^18^. Equivalent results were found in another work, however, a retrospective series of a few cases^22^.

In situations where curative lung resection is not feasible, such as in the presence of multiple unresectable metastases or pleural carcinomatous dissemination, systemic therapy should be established^10^. In the presence of unresectable lesions in the liver and resectable in the lung, if the Oslo criteria are met, which will be discussed later, transplantation may be a viable alternative^5^.

Several studies have been conducted in an attempt to treat associated liver and peritoneum metastases; however, the results of cytoreduction through peritoneal resection, complemented with hyperthermic chemotherapy and liver resection, are disappointing, with an overall 3-year survival of 40% and DFS of only 6%^12^.

### Lymph node may contraindicate resection

In relation to lymph nodes, during surgery, macroscopic invasion can be found in up to 10%, being classified as proximal (proper hepatic artery) and distant (celiac trunk/aorta). The presence of metastases in the liver pedicle historically has been associated with extremely poor outcomes (3% of 5-year survival). Nowadays, with new chemotherapy regimens, 18% 5-year survival can be expected versus 53% in patients without nodes. As for the distal lymph nodes, expectations are approximately 0%; therefore, the prognosis seems better when the patients have a satisfactory response, with regression of the disease after chemotherapy. It appears reasonable to consider surgery for patients with macroscopic invaded proximal nodes responding to chemotherapy^1^.

### What is the role of liver transplantation?

The treatment of colorectal cancer liver metastases remains fascinating and challenging due to multiple therapeutic options, as well as a constant evolution^6^. Through studies at the University of OSLO, a new perspective on unresectable cases has entered this complex arsenal of liver transplantation. Unlike the rest of the world, where there is a shortage of grafts available for allocation, this group has an excess of grafts, thus expanding the indications for transplantation and obtaining promising results. The very high rate of recurrence was alarming in the initial study; however, with the progression of treatment and constant evaluation of the results, OSLO criteria were defined as follows: maximum diameter of the largest tumor of ≤5.5 cm, stability of the disease with chemotherapy, the interval from primary tumor resection to transplant is ≥2 years, and CEA <80 ng/ml. Although the rate of DFS is alarming, the vast majority of relapses are isolated in the lung, leading to their resection and an overall 5-year survival of approximately 83%, which is much higher than that of other treatments^5^.

To date, no studies have been conducted on a large number of patients to validate the OSLO criteria, much less prospectively and randomly comparing their results with systemic chemotherapy or major liver resection. Given this situation, the increase in OS in the treatment of colorectal metastases must be evaluated, as the allocation of grafts in advanced colorectal cancer will reduce the demand for donors for other chronic liver pathologies.

## CONCLUSIONS

After the evolution of surgical treatment, the initial understanding of molecular biology in oncological pathologies has shown that cancer is a disease that requires multidisciplinary treatment. Knowledge of tumor behavior will help us better select the type, sequence, and individualized treatment of metachronous liver metastases.

## AUTHOR’S COMMENTS

Rene Adam: “We have had a consensus meeting of experts, medical oncologist, cardiologist and surgeon, and recently also European HPBA (Hepato-Pancreato-Biliary Association) consensus on that. And we keep the definition that synchronous is the diagnosis of the colon cancer or rectal cancer at the same time or up to one month after the diagnosis. This is synchronous, then come the early metachronous from one to 12 months, and then comes the late metachronous after 12 months of the diagnosis of the primary.”

Timothy M. Pawlik: “So I think that definition for academic purposes is a very good definition. I think in clinical practice, I tend to use more six months.”

Timothy M. Pawlik: “I would consider doing upfront surgery on because they have, you know, good biology if they have a low CEA and a solitary lesion on this really long indolent disease free period, consider doing upfront surgery on that patient.”

Paulo Herman: “We usually use as a guide, the clinical risk score, the Fong’s clinical risk score. And if the patient has less than three, we go to upfront surgery.”

Rene Adam: “The only problem is how all different teams are using the widespread use of such molecular biology profile because these were presented cost. Some time it’s not used widely. And we are all convinced that according to the mutation of gene and some disease, genetic profile, very important to take the decision and in the near future, will be probably very prominent to take decision”

Timothy M. Pawlik: “I agree with her (Maria from last Panel) and her comment that as soon as like BRAF mutated, that might wait a little bit longer, you know, we were talking about, like when to operate after we give systemic chemotherapy, because their prognosis is generally much worse.”

Timothy M. Pawlik: “Margin kRAS mutated patients, that, you know, we have some data to show that the prognostic significance of the margin isn’t as important because really, it’s the biology that’s driving your long term outcome. And I don’t know, this guy can help me with this technical difficulty. But, with the wild type, the biology is better. So kind of paradoxically, you know, that actually, you know, getting a wider margin might be more important.”

Rene Adam: “The prognosis is probably different in the presence of extrahepatic metastases, according to the type is. The best other lung, probably in terms of prognostic factor and the worst will be probably the peritoneum, I think unless being diffused, this should not be a contraindication.”

Paulo Herman: (About chemotherapy before surgery) “It’s the best way to understand biology, and we usually wait six months” 

Paulo Herman: “…has been shown many years ago that lymph nodes at the Highland 8,12, 13 Were not contraindication, we not only do chemotherapy, we wait at least six months of of stability or disappearance before going into surgery.”

Rene Adam: “I would say for lymph node of the hepatic pedicle It’s okay. So we don’t impair the survival of the patient. Of course, it’s a prognostic factors and survival is around 18% at five years, when we remove the lymph node of that particular article, but when it is retroperitoneal lymph node, at that time, the prognostic value of such type of location is much more important and up to recently, we had no long term survivors in those patients that we reserved from liver metastases with retroperitoneal lymphadenectomy.”

Alfred Kow: [on liver transplantation] We are doing as under the cover of clinical studies with living donor we don’t accept patient with synchronous tumor, unless they have a very good window period where the primary is removed, and they still remain stable and liver only disease. I think we generally wait for at least more than one year, if not two years for the interval between the primary, the molecular profiling is important. So we study the KRAS / BRAF mutation very carefully, also the response to the chemotherapy, we generally want them to at least have seen two lines of chemotherapy. And it’s clearly not resectable. Because if the response to chemotherapy, resectable resection should be the primary consideration before transplantation.”

Rene Adam: “Transplantation for very selected patient may provide an 80% overall survival rate that five years and that some patient may really be cured by liver transplantation, on the lung is this is an indolent disease and the survival still remain even with reference by lung metastases 72% At five years, when the patient recurrent deliver it’s very, very, very bad prognosis with no patient alive at more than two years from transplantation. So, what we see is the overall survival 80%, now with very good selective. I would say criteria like you can see here in the randomized study that we conduct, we have conducted very good selection, good overall survival, but still, I would say this is with survival, which is around 50%. And so we should refine probably, again, this is the selection of the patient but today, very obviously, we can cure patients with liver transplantation.”

Olivier Soubrane: “Another problem is the so called little gap, which is common for all transplant oncology, meaning the number of liver graft available versus the number of patients waiting for transplant waiting list. When you look at the United States, it’s about 9000 liver transplants a year *versus* 14,000 new patient put on the waiting list. I think indications selection of patients is extremely important, but we must find new sources of liver graft to the liver gap increased too much.”
